# Symptoms of Problem Gambling Among US Adults Who Wager on Sports

**DOI:** 10.1001/jamanetworkopen.2022.39670

**Published:** 2022-10-31

**Authors:** Joshua B. Grubbs, Shane W. Kraus

**Affiliations:** 1 Bowling Green State University, Bowling Green, Ohio; 2 University of Nevada, Las Vegas, Las Vegas, Nevada

## Abstract

This survey study assesses levels of problem-gambling risk across 4 categories of sports-related wagering among US adults.

## Introduction

Recent US Supreme Court rulings, increased promotion of fantasy and daily fantasy sports,^1^ and the rise of e-sports^2^ have all fueled new avenues for US individuals to gamble on sports.^3^ Even so, research in this domain has lagged in the US.^4^ To address this lag, we examined 4 categories of sports-wagering behaviors: (1) general sports wagering, (2) paid fantasy league play, (3) daily fantasy league play, and (4) e-sports wagering.

## Method

In March of 2022, US adults matched and weighted for US norms for age, gender, education, census region, and race and ethnicity as of the 2019 American Community Survey^5^ and an oversample of sports-wagering adults in the US, were recruited via YouGov. Full information about this survey and comprehensive demographics data are available online (eTable and eAppendix in the Supplement). Survey results are reported in accordance with the American Association for Public Opinion Research (AAPOR) reporting guideline for nonprobability internet panels. This work was approved by Bowling Green State University institutional review board.

Participants reported their past 12-month engagement with the aforementioned 4 categories of sports wagering. Gamblers completed the Problem Gambling Severity Index^6^ (PGSI; range = 0-27; mean [SD], 3.78 [6.04]; α = .95; ω = 0.95), resulting in categorizations of scores^6^ so that respondents scoring 0 (47% of gamblers in sample) were categorized as no-risk gamblers, 1 to 2 (20.6%) as low risk, 3 to 7 (12.3%) as moderate risk, and 8 and above (20.1%) as high risk.

Statistical analyses were conducted in SPSSv25 and the *stats* package in R statistical software (R Project for Statistical Computing). We included gender, age, race, education, income, legality of sports wagering in state of residence, and religiousness as covariates in analyses. We conducted binary logistic regressions, regressing all forms of sports-betting on demographic covariates, and a multinomial logistic regression regressing PGSI score category on demographic covariates and binary indicators of past 12-month sports wagering. Two-tailed *P* < .05, after Holm correction for multiple comparison, was considered statistically significant.

## Results

The national, census-matched survey consisted of 2806 participants (mean [SD] age, 48.9 [17.2] years; 1365 men [48.6%]; 340 Black individuals [12.1%] , 460 Hispanic individuals [16.4%], 1762 White individuals [62.8%]; response rate = 87.6%). The oversample of sports gamblers consisted of 1557 participants (mean [SD] age, 41.7 [15.3] years; 1043 men [67.0%]; 224 Black individuals [14.4%], 262 Hispanic individuals [16.8%], 830 White individuals [53.3%]; response rate, 78.7%). Weighted estimates in our census-matched sample found that 17.2% (n = 485) had wagered on sports in their lifetime, with 6.2% (n = 175) endorsing past-year play. Regarding e-sports, 9.1% (n = 256) endorsed lifetime history, with 4.1% (n = 114) endorsing past-year play. Regarding paid fantasy sports, 12.9% (n = 364) endorsed lifetime history, with 5.9% (n = 166) endorsing past-year play. Regarding daily fantasy league play, 10.3% (n = 287) endorsed lifetime history, with 4.2% (n = 117) endorsing past-year play.

In the combined sample, binary logistic regressions revealed that, for all forms of sports-wagering involvement, being male, younger, more religious, of higher income, and residing in a state where sports wagering is legal were associated with greater likelihood of play (Table 1).

**Table 1.** Logistic Regressions and Associated Odds Ratios Estimating Past 12-Month Engagement in Various Types of Sports Wagering Based on Demographic Variables

	Odds ratio (95% CI)				
	Any sports wagering	Paid fantasy leagues	Daily fantasy leagues	General sports wagering	E-sports wagering
Male gender^a^	2.05 (1.78-2.36)	2.17 (1.84-2.56)	2.10 (1.74-2.54)	1.81 (1.55-2.12)	1.55 (1.28-1.88)
Age, y	0.97 (0.97-0.98)	0.97 (0.97-0.98)	0.96 (0.96-0.97)	0.99 (0.98-0.99)	0.96 (0.96-0.97)
Religiousness	1.18 (1.09-1.28)	1.16 (1.06-1.27)	1.37 (1.4 1.52)	1.09 (1.00-1.19)	1.63 (1.46-1.82)
Race^b^					
American Indian or Alaska Native	2.05 (1.14-3.70)	2.30 (1.33-3.96)	1.74 (0.97-3.11)	2.29 (1.34-3.91)	2.10 (1.18-3.75)
Asian	1.08 (0.73-1.61)	1.09 (0.72-1.66)	1.28 (0.82-2.02)	0.84 (0.54-1.29)	1.25 (0.77-2.02)
Biracial	0.72 (0.45-1.17)	0.50 (0.27-0.94)	0.53 (0.26-1.10)	0.92 (0.55-1.54)	1.12 (0.61-2.07)
Black	1.11 (0.88-1.38)	0.98 (0.76-1.27)	1.32 (1.00-1.75)	0.87 (0.68-1.12)	1.21 (0.90-1.61)
Hispanic	0.78 (0.62-0.98)	1.01 (0.78-1.31)	1.03 (0.77 1.38)	0.83 (0.63-1.07)	1.04 (0.77-1.41)
Middle Eastern	6.37 (1.79-22.64)	0.97 (0.35-2.68)	1.41 (0.50-3.98)	2.38 (0.95-6.00)	1.17 (0.40-3.39)
Other^c^	0.94 (0.56-1.58)	1.12 (0.63-1.99)	1.50 (0.80-2.82)	1.18 (0.69-2.03)	1.29 (0.66-2.53)
Education^d^					
No high school diploma	0.71 (0.43-1.19)	0.66 (0.34-1.29)	0.40 (0.16-1.02)	0.70 (0.38-1.29)	0.96 (0.49-1.89)
Some college	1.28 (1.04-1.59)	1.20 (0.93-1.56)	1.17 (0.87-1.56)	1.14 (0.90-1.46)	0.81 (0.59-1.11)
2 Year degree	1.52 (1.18-1.96)	1.38 (1.03-1.85)	1.32 (0.94-1.84)	1.45 (1.10-1.92)	1.27 (0.91-1.77)
4 Year degree	1.45 (1.18-1.79)	1.45 (1.14-1.85)	1.24 (0.94-1.64)	1.37 (1.09-1.72)	1.08 (0.82-1.44)
Postgraduate	1.30 (1.02-1.66)	1.20 (0.91-1.58)	1.53 (1.12-2.10)	1.36 (1.05-1.77)	0.97 (0.70-1.34)
Income	1.12 (1.10-1.15)	1.09 (1.06-1.11)	1.07 (1.04-1.10)	1.08 (1.05-1.10)	1.06 (1.03-1.09)
Legality	2.86 (2.48-3.29)	2.12 (1.80-2.49)	2.40 (1.99-2.89)	2.75 (2.36-3.21)	2.59 (2.13-3.16)
Cox and Snell *R^2^*	0.19	0.12	0.11	0.11	0.10
Nagelkerke *R^2^*	0.25	0.18	0.19	0.16	0.18

^a^ Gender coded as men = 1, Women/Nonbinary/Other = 0.

^b^ White participants used as the reference group.

^c^ Specific races and ethnicities included in this category were not specified.

^d^ Participants who graduated high school only used as the reference group.

With regards to problem gambling scores, beyond demographic controls, engagement in general sports betting, daily fantasy play, and e-sports wagering were each associated with being categorized as a moderate-risk or-high-risk gambler, although general fantasy sports play was not (Table 2).

**Table 2.** Multinomial Logistic Regression Estimating Category of Problem Gambling Severity Index Score Based on Demographic Controls and Sports-Wagering Status With No-Risk Gamblers as the Reference Group

	Odds ratio (95% CI)		
	Low risk	Moderate risk	High risk
Male gender	1.24 (1.00-1.52)	1.08 (0.84-1.39)	0.83 (0.65-1.06)
Age, y	1.00 (0.99-1.01)	0.98 (0.97-0.99)	0.93 (0.92-0.94)
Religiousness	1.25 (1.11-1.41)	1.22 (1.05-1.41)	1.93 (1.66-2.24)
Income	0.96 (0.93-0.99)	0.92 (0.89-0.96)	0.99 (0.96-1.03)
Legality	1.04 (0.84-1.27)	1.44 (1.12-1.86)	1.29 (1.01-1.66)
General sports bettor	1.81 (1.45-2.25)	1.32 (1.01-1.73)	1.60 (1.24-2.07)
Fantasy sports bettor	1.03 (0.80-1.33)	1.28 (0.95-1.72)	1.14 (0.86-1.5)
Daily fantasy sports bettor	1.24 (0.92-1.67)	1.49 (1.07-2.08)	2.59 (1.93-3.48)
E-sports bettor	1.34 (0.99-1.81)	2.68 (1.96-3.66)	2.84 (2.12-3.81)
Race^a^			
American Indian and Alaska Native	1.63 (0.60-4.46)	1.34 (0.40-4.55)	6.67 (2.66-16.76)
Asian	1.74 (0.87-3.48)	2.93 (1.47-5.84)	3.61 (1.90-6.85)
Biracial	1.21 (0.61-2.41)	0.44 (0.13-1.48)	2.06 (1.00-4.21)
Black	1.08 (0.77-1.50)	1.02 (0.68-1.53)	2.28 (1.60-3.24)
Hispanic	0.96 (0.67-1.37)	1.28 (0.86-1.91)	2.46 (1.72-3.51)
Middle Eastern	1.86 (0.11-30.25)	5.30 (0.47-59.69)	17.04 (2.16-134.42)
Other^b^	0.49 (0.20-1.21)	0.28 (0.07-1.20)	1.72 (0.80-3.67)
Education^c^			
No high school diploma/	2.15 (1.1-4.17)	1.1 (0.44-2.72)	2.37 (1.11-5.09)
Some college	1.14 (0.84-1.54)	0.94 (0.65-1.34)	0.72 (0.49-1.06)
2 y degree	1.26 (0.87-1.82)	1.06 (0.69-1.64)	1.01 (0.66-1.55)
4 y degree	0.99 (0.73-1.34)	0.59 (0.40-0.85)	0.59 (0.41-0.85)
Postgraduate	0.92 (0.65-1.32)	0.72 (0.47-1.11)	0.54 (0.36-0.83)
Cox and Snell *R^2^*^d^	0.317		
Nagelkerke *R^2^*^d^	0.344		

^a^ White participants used as the reference group.

^b^ Specific races and ethnicities included in this category were not specified.

^c^ Participants who graduated high school only used as the reference group.

^d^ This *R^2^* value is for the total analysis, so it applies to the information across all columns in this row.

## Discussion

Sports wagering is a niche, but not rare, recreational activity among US adults, with younger men with greater family income being especially prone to wager. E-sports wagering, daily fantasy league play, and general sports wagering are each associated with greater symptoms of problem gambling. Although sports wagering is likely a safe and reasonable recreational activity for many people, our findings suggest that individuals who wager on sports may be at higher risk of gambling-related problems.

A limitation of this study is that it was based on cross-sectional self-report in a nonprobability sample, demonstrating a need for more rigorous studies of these phenomena. As access to sports wagering rises in the US, it is crucial that psychiatric and mental health communities consider sports wagering in future research and in clinical care.

## Notes

Supplement. **eTable.** Demographics of Total Sample, Sports Wagering Individuals, and Non-Sports Wagering Individuals **eAppendix.** Sports Wagering in the United States
