# Supplementary material for: Symptoms of Problem Gambling Among US Adults Who Wager on Sports
Source: JAMA Netw Open. 2022 Oct 31;5(10):e2239670. doi: 10.1001/jamanetworkopen.2022.39670 (PMC9623440; doi:10.1001/jamanetworkopen.2022.39670)
Supplement: Supplement. — eTable. Demographics of Total Sample, Sports Wagering Individuals, and Non-Sports Wagering Individuals eAppendix. Sports Wagering in the United States [file jamanetwopen-e2239670-s001.pdf]

Supplemental Online Content

Grubbs JB, Kraus SW. Symptoms of problem gambling among US adults who wager on sports. *JAMA Netw Open.* 2022;5(10):e2239670.
doi:10.1001/jamanetworkopen.2022.39670

eTable. Demographics of Total Sample, Sports-Wagering Individuals, and Non-Sports Wagering Individuals
eAppendix. Sports Wagering in the United States

This supplemental material has been provided by the authors to give readers additional information about their work.

© 2022 Grubbs JB et al. *JAMA Network Open.*

eTable. *Demographics of Total Sample, Sports Wagering Individuals, and Non-Sports Wagering Individuals*

	Total Sample	Live Action Sports	Paid Fantasy Leagues	Daily Fantasy Leagues	E-sport Wagering	Any Sports Wagering	No Sports Wagering
N	4363	1093	962	703	602	1812	2551
Mean Age (SD)	49.56 (16.15)	46.36 (14.78)	44.35 (14.04)	42.48 (13.83)	42.36 (14.91)	45.85 (15.06)	52.21 (16.38)
Gender							
Men	51.4%	65.4%	68.7%	69.0%	63.5%	63.6%	42.8%
Women	46.4%	32.2%	28.5%	28.0%	32.75	33.1%	55.9%
Other	2.2%	2.4%	2.8%	3.0%	3.8%	3.3%	1.3%
Race							
White	66.7%	68.8%	67.0%	63.2%	60.1%	67.4%	66.2%
Black	12.0%	10.6%	11.0%	13.7%	14.1%	11.9%	12.1%
Hispanic	11.3%	9.1%	10.4%	10.4%	11.5%	9.4%	12.7%
Asian	3.2%	3.0%	3.8%	4.3%	4.2%	3.6%	2.9%
Native American	16.%	3.1%	3.5%	3.7%	4.5%	2.6%	0.9%
biracial	2.4%	2.3%	1.6%	1.6%	2.7%	2.1%	2.6%
Middle Eastern	2.2%	0.9%	0.6%	0.9%	0.8%	2%	2.4%
Other	0.5%	2.1%	2.0%	2.4%	2.2%	1.0%	0.2%
Education							
No High School Diploma or equivalent	2.9%	1.3%	1.2%	.9%	1.8%	1.5%	3.8%
HS Graduate	26.8%	19.1%	18.4%	18.1%	21.6%	19%	32.4%
Some College	19.2%	16.7%	17.0%	16.8%	14.3%	18%	20%
2 year degree	10.5%	11.7%	11.3%	11.5%	13.0%	11.5%	9.8%
4 year degree	24.9%	31.5%	33.1%	30.6%	31.7%	31%	20.7%
Post-grad	15.7%	19.7%	18.9%	22.2%	17.6%	19%	13.3%
Median Income	\$60,000-\$69,999	\$70,000-\$79,999	\$70,000-\$79,999	\$70,000-\$79,999	\$70,000-\$79,999	\$70,000-\$79,999	\$50,000-\$59,999

eAppendix. Sports Wagering in the United States

Full information for this study's survey can be found online at https://osf.io/wqtrf/.

© 2022 Grubbs JB et al. *JAMA Network Open.*
